# High-throughput quantification of microbial-derived organic acids in mucin-rich samples via reverse phase high performance liquid chromatography

**DOI:** 10.1099/jmm.0.001708

**Published:** 2023-06-09

**Authors:** Alex R. Villarreal, Sarah K. Lucas, Joshua R. Fletcher, Ryan C. Hunter

**Affiliations:** 1 Department of Microbiology & Immunology, University of Minnesota, 689 23rd Avenue SE, Minneapolis, MN 55455, USA

**Keywords:** amino acids, glycoproteins, high performance liquid chromatography, mucin, organic acids, reverse phase liquid chromatography, targeted metabolomics

## Abstract

Organic acids (short chain fatty acids, amino acids, etc.) are common metabolic byproducts of commensal bacteria of the gut and oral cavity in addition to microbiota associated with chronic infections of the airways, skin, and soft tissues. A ubiquitous characteristic of these body sites in which mucus-rich secretions often accumulate in excess, is the presence of mucins; high molecular weight (HMW), glycosylated proteins that decorate the surfaces of non-keratinized epithelia. Owing to their size, mucins complicate quantification of microbial-derived metabolites as these large glycoproteins preclude use of 1D and 2D gel approaches and can obstruct analytical chromatography columns. Standard approaches for quantification of organic acids in mucin-rich samples typically rely on laborious extractions or outsourcing to laboratories specializing in targeted metabolomics. Here we report a high-throughput sample preparation process that reduces mucin abundance and an accompanying isocratic reverse phase high performance liquid chromatography (HPLC) method that enables quantification of microbial-derived organic acids. This approach allows for accurate quantification of compounds of interest (0.01 mM – 100 mM) with minimal sample preparation, a moderate HPLC method run time, and preservation of both guard and analytical column integrity. This approach paves the way for further analyses of microbial-derived metabolites in complex clinical samples.

## Introduction

Mucin glycoproteins, the primary macromolecular constituent of mucus, range from 1 to 20 megadaltons in size and consist of a threonine-, serine-, proline-, and cysteine-rich polypeptide backbone extensively decorated by O-linked glycans [1,3]. Secreted by mucosal epithelial cells [4], mucins aggregate through the formation of disulfide bonds and non-covalent interactions to form a hydrogel that lends mucus its characteristic viscosity and structure [4,5]. Mucins line all non-keratinized epithelial surfaces and are vital in the maintenance of the ocular, oral, respiratory, gastrointestinal (GI), and reproductive systems [6], as they serve a variety of barrier and innate immunity functions [6,7]. Conversely, disruptions, modifications, or aberrant production of the mucus layer can lead to chronic infection (e.g. cystic fibrosis, chronic rhinosinusitis) or dysbiotic bacterial community assembly (e.g. in the large intestine) [8].

In the case of cystic fibrosis (CF), mutations in the gene encoding the cystic fibrosis transmembrane regulator (CFTR) protein lead to ionic imbalance and accumulation of viscous, dehydrated mucus in the airways [9,10]. Mucus accretion not only impairs respiratory function through physical obstruction and eliciting inflammation, but also impairs mucociliary clearance – a vital function of innate immunity that removes exogenous microbiota [11,12]. Ultimately, this results in the development of chronic bacterial infection by *Pseudomonas aeruginosa, Staphylococcus aureus*, and other airway pathogens, which further deteriorate respiratory function over time [13,14]. Interestingly, unlike many GI microbiota, canonical airway pathogens inefficiently utilize mucins as a nutrient source [14]. Instead, their growth is thought to be supported by other host-derived nutrients and/or secondary metabolites produced via degradation and mixed-acid fermentation of mucins by co-colonizing bacteria [14]. Among these metabolites, organic acids (e.g. short chain fatty acids, amino acids) are most abundant [14,15] and are known to shape the growth, physiology, and antibiotic susceptibility of airway pathogens [14,16].

Quantification of organic acids in clinical samples and bacterial culture supernatants is commonly accomplished by analyte separation via liquid or gas chromatography followed by via mass spectrometry [14,17]. However, these workflows can be problematic for quantification of organic acids in mucus-rich samples due to the HMW and gelling properties of mucins. Their intrinsic properties leave mucins incompatible with many chromatography methods as they preclude the use of 1D and 2D gel approaches and can obstruct analytical separation columns and chromatography instruments [18,19]. To overcome these complications, mucins can be depleted via acid hydrolysis [20,21] or organic acids can be extracted from mucin-rich samples via liquid-liquid or solid phase extraction prior to analysis [14,22]. However, extraction methods inherently result in variable reduction of target analyte concentrations which must be accounted for in final quantification, introducing the potential for error and reduced accuracy. Samples can also be outsourced to laboratories specializing in metabolomics [23], but this approach can be low-throughput, time consuming, and cost-prohibitive.

We sought to overcome these challenges by developing a high-throughput sample preparation approach capable of depleting mucin content with minimal impact on organic acid concentrations. We then sought to develop a compatible high performance liquid chromatography (HPLC) method able to separate and quantify organic acids of interest without the need for subsequent mass spectrometry validation. Here we describe the validation of our workflow that accomplishes these objectives and offers a high-throughput, cost-effective method for the quantification of microbial-derived metabolites across a range of mucin-rich environments.

## Methods

### Chemicals

l-isoleucine, l-leucine, l-phenylalanine, and l-valine were purchased from Alfa Aesar (Massachusetts, USA). l-lysine and l-proline were purchased from Sigma-Aldrich (Missouri, USA). l-methionine and l-threonine were purchased form Arcos Organics (New Jersey, USA). Sodium acetate, sodium pyruvate, and citric acid were purchased from Fisher Scientific (Massachusetts, USA). Anhydrous sodium sulphate, sodium butyrate, sodium propionate, sodium l-lactate, sodium formate, sodium hexanoate, and methanesulfonic acid were purchased from Sigma-Aldrich (Missouri, USA). Sodium succinate was purchased from Fluka (Neu-Ulm, Germany). Anhydrous acetonitrile was purchased from VWR (Pennsylvania, USA).

All standards were prepared using the filtrate of a minimal mucin medium (see below) run through a Pierce polyethersulfone (PES) 3000 MWCO 0.5 ml protein concentrator (Thermo Scientific, Massachusetts, USA). Then 10 mM or 1 M stock solutions of all organic acids were used to produce a range of standards (0.01 mM-100 mM). All standards were filtered through a 0.22 µm PES centrifuge filter (Thermo Scientific) prior to analysis.

### Media preparation

A minimal mucin medium (MMM) was prepared as previously described [14] with modifications. Briefly, type III porcine gastric mucin (PGM) (Sigma-Aldrich) was added to ddH_2_O at a concentration of 30 g l^−1^ prior to autoclaving at 121°C for 15 min and centrifugation at 15 000 ***g*** for 1 h. Supernatant was carefully removed, avoiding solids, and sequentially filtered through PES membranes (3 µm, 1 μm, 0.45 µm, 0.22 µm) (Merck Millipore, Massachusetts, USA) using a peristaltic pump. Filtrate was transferred into 20 µm regenerated cellulose (RC) dialysis tubing (Fisher Scientific) for two, 2 h incubations in ddH_2_O at room temperature, followed by overnight incubation at 4°C. ddH_2_O was replaced between each incubation. Post dialysis, ddH_2_O was added to the mucin solution at a 1 : 1 ratio and was buffered with 50 mM KH_2_PO_4_ and 150 mM NaCl. Then 1 mM MgSO_4_ and a vitamin and mineral mix [14] were added to the buffered mucin solution and filtered through a 0.22 µm bottle top PES filter (Foxx Life Science, New Hampshire, USA). Mucin content was determined using FPLC as described below. Complete medium was stored at −20°C for immediate use (<2 weeks) or −80°C for long-term storage (>2 weeks).

### Fast protein liquid chromatography (FPLC)

FPLC analysis was performed on a ÄKTA Pure instrument with compatible ALIAS autosampler equipped with a 10/200 mm Tricorn column packed with Sepharose CL-2B agarose gel filtration base matrix (Cytiva; Massachusetts, USA). A minimum of 700 μl per sample was filtered through 0.22 µm PES centrifuge filters before transfer into 2 ml crimp top glass vials with pierceable septa (Thermo Scientific). Then 500 μl of each sample filtrate was injected via the autosampler and subjected to a 48 min isocratic run method at a flow rate of 0.4 ml min^−1^ with a mobile phase consisting of 50 mM phosphate buffer and 150 mM NaCl brought to a pH of 7.2 using 1 N HCl.

### Clinical sample collection

Expectorated mucus was collected from adult subjects with cystic fibrosis at the University of Minnesota Adult CF Centre as previously described [14].

### Cultivation of microorganisms

Bacterial cultivation was performed in an anaerobic chamber (Coy Laboratory Products, Michigan, USA) containing a gas mixture of 90 % N_2_, 5 % H_2_, and 5 % CO_2_. All media was allowed to de-gas and equilibrate in the chamber for more than 12 h before use. *Streptococcus gordonii* DL1, *Prevotella melaninogenica* ATCC25845*, Veillonella parvula* ATCC10790*,* and *Fusobacterium nucleatum* ATCC26686, were each maintained on Brain Heart Infusion (BHI) agar containing hemin and vitamin K and used to inoculate 3 ml of BHI broth containing hemin and vitamin K and MMM at a 1 : 1 ratio. Following a 48 h incubation at 37°C, cultures were passaged into 3 ml of MMM and incubated at 37°C for another 48 h before centrifugation at 10 000 ***g*** for 2 min. Pellets were washed twice with MMM and used to inoculate experimental cultures for downstream HPLC analysis. Likewise, an anaerobic microbial community was enriched from CF mucus. To do so, 1 ml of PBS was added to a clinical sample, mechanically homogenized, and was used to inoculate 3 ml culture of 1 : 1 BHI : MMM. Following a 48 h incubation, the culture was passaged into 3 ml of MMM and incubated at 37°C for another 48 h. This was repeated for a total of four passages before the final culture was used to generate a 20 % glycerol stock and supernatant for downstream HPLC analysis.

### Reverse phase high performance liquid chromatography (RP-HPLC)

HPLC was used to quantify concentrations of organic acids (Table 1) and amino acids (Table 2). Bacterial cultures grown in MMM were first centrifuged at 4 000 ***g*** for 10 min. Supernatants were then filtered through 0.22 µm PES centrifuge filters. Supernatants were then filtered through a 0.5 ml 3000 MWCO PES protein concentrator (Thermo Scientific) to remove HMW mucins. For analysis of raw samples, 1 ml of PBS was added to sputum before homogenization. A 1 ml aliquot was then removed and subjected to the sample preparation method described above.

**Table 1.** Organic acids and their respective detection parameters corresponding to pure standards in minimal mucin media (MMM) as determined by (HPLC)

Metabolite	Classification	Average Retention Time (min)	Standard Range (mM)	R^2^	PES Filter Yield (%)
Acetate	Short-chain fatty acid	3.60	0.1–100	1	99.94
Butyrate	Short-chain fatty acid	17.75	0.1–100	1	96.43
Citrate	Tricarboxylic acid	4.60	0.1–100	0.99	82.35
Formate	Monocarboxylic acid	2.65	1–100	0.99	102.00
Lactate	Alpha-hydroxy acid	3.4	0.1–100	0.99	93.61
Propionate	Short-chain fatty acid	7.30	0.1–100	0.99	91.93
Pyruvate	Keto acid	2.80	0.1–100	0.99	83.94
Succinate	Dicarboxylic acid	5.35	0.1–100	0.99	115.82

**Table 2.** Amino acids and their respective detection parameters corresponding to pure standards in minimal mucin media (MMM) as determined by HPLC

Metabolite	Classification	Average Retention Time (min)	Standard Range (mM)	R^2^	PES Filter Yield (%)
Isoleucine	Branched-chain, nonpolar, aliphatic amino acid	5.70	0.01–10	0.99	97.70%
Leucine	Branched-chain, nonpolar, aliphatic amino acid	6.15	0.01–10	0.99	105.57%
Lysine	Polar, positively charged amino acid	2.00	0.01–10	0.99	116.15%
Methionine	Nonpolar, aliphatic amino acid	3.90	0.01–10	0.99	79.94%
Phenylalanine	Nonpolar, aromatic amino acid	14.45	0.01–10	1	86.35%
Proline	Polar, uncharged amino acid	2.35	0.01–10	0.99	125.00%
Threonine	Polar, uncharged amino acid	2.15	0.01–10	0.99	78.56%
Valine	Branched-chained, nonpolar, aliphatic amino acid	3.10	0.01–10	0.99	100.87%

Filtrates were analysed using a Dionex UltiMate 3000 HPLC system operated by Chromeleon software (v.7.0, Thermo Fisher) comprised of a compatible rapid separation (RS) pump, autosampler, column oven, fluorescence detector, RS diode array, and fraction collector (Thermo Fisher). This instrument was equipped with an Acclaim organic acid 5 µm 120A° 4.0×250 mm column and accompanying guard column (Thermo Fisher). Analyte separation was achieved using an isocratic run method with a mobile phase of 100 mM NaSO_4_ (pH adjusted to pH 2.6 using CH_3_SO_3_H). This method consisted of an 8 min equilibration step and subsequent 24 min static flow at a rate of 1 ml min^−1^. A total of 6 µl of sample was used per run and injected into the system via the autosampler, with a brief washing of the injection needle with 10 % CH_3_OH before and after each injection. Column oven temperature was maintained at 30°C. The RS diode array was configured to collect UV readings at the wavelengths of 210 nm with default frequency.

Chromeleon software (v.7.0) was used to view and process raw data. Raw chromatograms were stacked and offsets removed to compare standard runs and perform an initial quality control of each run sequence. Quantitative processing methods were created for each target analyte. The integrated Cobra Wizard was used to gate and smooth peaks of interest on a single standard of intermediate concentration (1–10 mM) for a given analyte. To ensure gating accuracy, each sample was manually checked and the area under the curve (AUC) recorded for later processing in Prism 9.0 (GraphPad). Cobra wizard was run with default settings.

### Concentrator analyte recovery

MMM was filtered through a 3 000 MWCO PES protein concentrator to remove HMW mucins. The mucin-depleted flow-through was then used to prepare 10 mM standards of organic acids (Table 1) and 1 mM standards of amino acids (Table 2) – preventing the analytes from coming in direct contact with the filter. Identical standards were then prepared in unfiltered MMM before being filtered through 3 000 MWCO PES protein concentrators – exposing the analytes to the filter. Paired standards of each analyte were then run back-to-back on the HPLC using methods described above. Chromeleon 7.0 and Prism 9.0 (GraphPad) were used to calculate concentrations of analyte in each sample. Analyte recovery was determined by comparison of change in analyte concentration between the two methods of preparation. To compare filter-based analyte recovery to a more common extraction approach, 10 mM standards of acetate, butyrate, lactate, and propionate were first prepared in MMM before undergoing a liquid-liquid extraction as previously described [24].

### Analytical column wear

A 10 mM standard of acetate was run on a new Acclaim organic acid (OA) analytical column to measure analyte peak properties under ideal column conditions. The same analytical column was then subjected to a total of 145 runs consisting of standards, bacterial supernatants, and clinical sample supernatants, prior to a final run of the same 10 mM acetate standard. A column cleaning protocol was performed as recommended by the manufacturer [25], consisting of flushing the column with 10 column volumes of 0.22 µm filtered ddH_2_O, then 20 column volumes of 100 % acetonitrile, then another 10 column volumes of 0.22 µm filtered ddH2O, before a final equilibration of the column with 15 column volumes of 100 mM Na^2^SO_4_ (pH=2.65). Following this, the same 10 mM acetate standard was run again. The accompanying guard column was not replaced prior to cleaning. Data were processed and compared as described above.

## Results & discussion

### Depletion of high molecular weight mucins

**Fig. 1.**
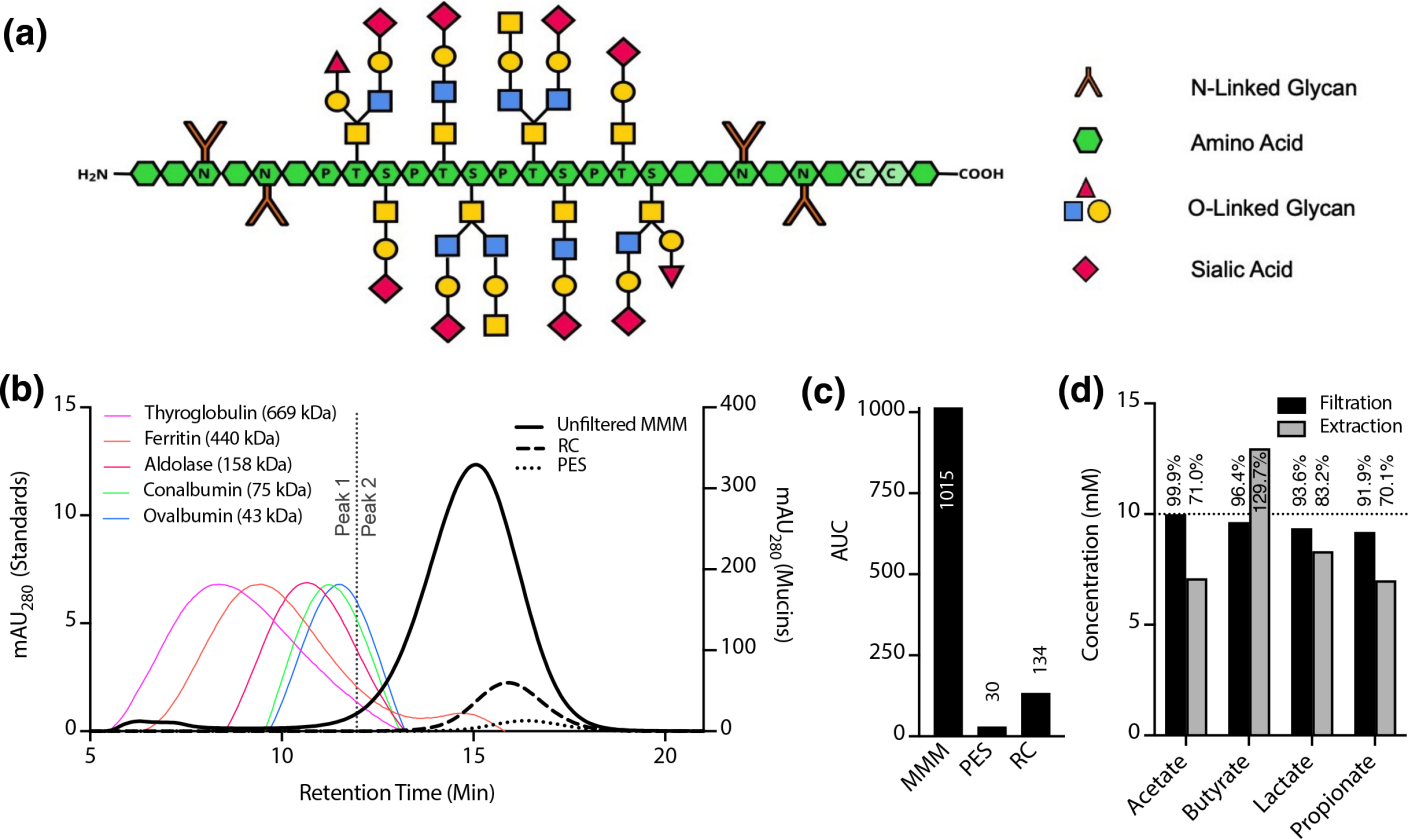
Mucin glycoproteins can be efficiently depleted from mucin-rich samples via filtration. (**a**) Chemical structure of a mucin glycoprotein. (**b**) Fast protein liquid chromatography (FPLC) chromatogram conveying relative size and concentration of protein standards (left y-axis) and minimal mucin media (MMM) (right y-axis) subjected to mucin depletion using 3000 MWCO regenerated cellulose (RC) or 3000 MWCO polyethersulfone (PES) protein concentrators. Ovalbumin (43 kDa) RT=11.55, conalbumin (75 kDa) RT 11.22, aldolase (158 kDa) RT=10.60, ferritin (440 kDa) RT=9.17, and thyroglbulin (669 kDa) RT=8.17. (**c**) Area under the curve (AUC) values conveying relative mucin concentration in MMM pre- and post-filtration. (**d**) Yield (mM) of 10 mM standards of acetate, butyrate, lactate, and propionate in MMM after mucin depletion (filtration) and liquid-liquid extraction.

The size and intrinsic properties of mucin (Fig. 1a) can interfere with most analytical chromatography methods. Thus, we sought to develop an approach to deplete mucins from bacterial cultures and human-derived clinical samples without reducing concentrations of organic acids of interest. To do so, MMM was filtered through 3 000 MWCO polyethersulfone (PES) and regenerated cellulose (RC) protein concentrators and analysed via FPLC. Unfiltered MMM and a series of high molecular weight (HMW) protein standards (Cytiva) were used to establish the relation between protein size (kDa) and column retention time (RT) (Fig. 1b, d). Chromatograms shown demonstrate that full depletion of HMW mucins (peak 1, RT 5–12 min) was achieved using both filtration methods. As determined by peak area under curve (AUC), low molecular weight (LMW) mucins (peak 2, RT 12–20 min) were reduced by ~97 and 86 % by PES and RC filtration, respectively, demonstrating the efficacy of our protocol. We then determined analyte recovery post-filtration to ensure no significant loss of metabolite concentrations (Tables1  2). Then 10 mM solutions of four representative analytes (acetate, butyrate, propionate, and lactate) were also subjected to a more conventionally used liquid-liquid extraction approach for comparison (Fig. 1d). In each case, PES filtration resulted in analyte recovery percentages near 100 %, demonstrating improvement over standard liquid extraction approaches.

### Quantification of organic acids in mucin-rich specimens

**Fig. 2.**
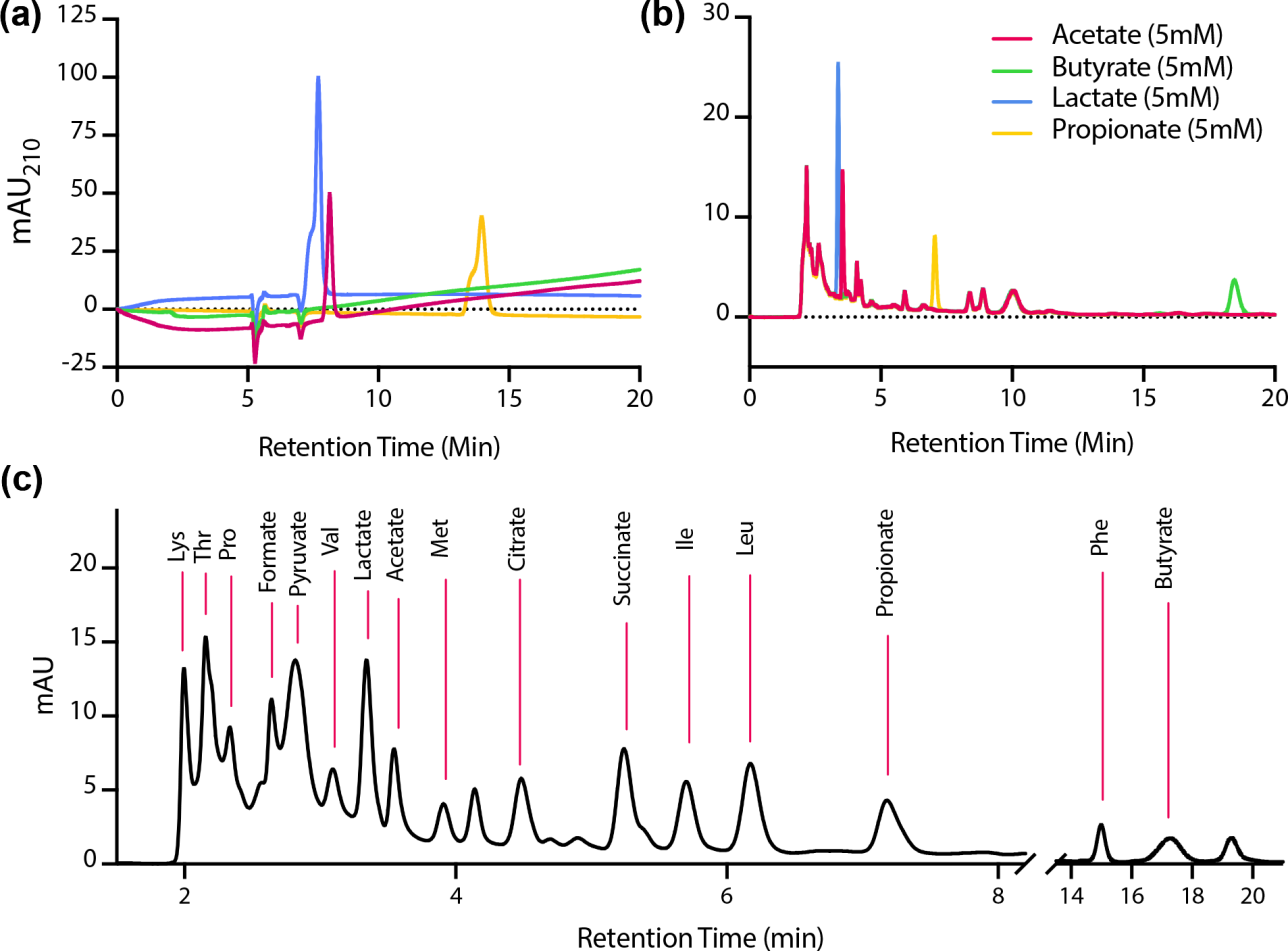
Mucin depletion improves analyte resolution and eliminates peak aberrations. (**a**) High performance liquid chromatography (HPLC) chromatogram of 5 mM standards of acetate, butyrate, lactate, and propionate in minimal mucin media (MMM) without a mucin depletion step. (**b**) HPLC chromatogram of the same standards using the same run method but with the inclusion of a mucin depletion step. Each standard was run separately. (**c**) HPLC chromatogram of a mixed standard solution of amino acids (0.938 mM, Table 2) and other organic acids (3.125 mM, Table 1).

**Fig. 3.**
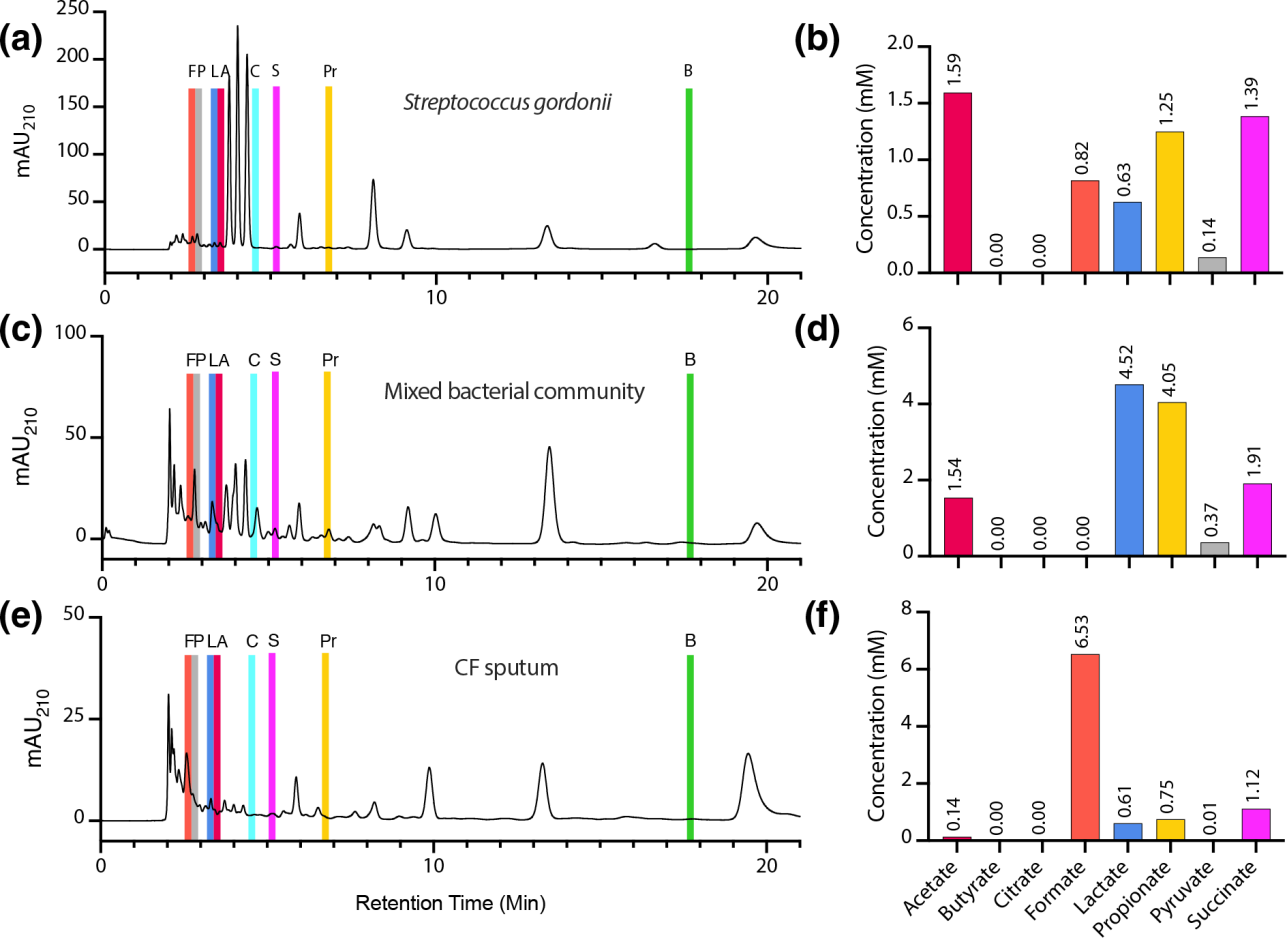
HPLC workflow allows for the quantification of organic acids in mucin-rich sample types. (**a, b**) HPLC chromatogram and corresponding organic acid profile of supernatant generated by *Streptococcus gordonii* grown anaerobically in MMM. (**c, d**) HPLC chromatogram and organic acid profile of supernatant generated by a mixed anaerobic bacterial community derived from a clinical CF sputum sample. (**e, f**) HPLC chromatogram and corresponding organic acid profile of a raw clinical CF sputum sample. F=formate, P=pyruvate, L=lactate, A=acetate, C=citrate, S=succinate, Pr=propionate, B=butyrate.

To further demonstrate the efficacy of filtration-based sample preparation, 5 mM standards of representative organic acids (acetate, butyrate, lactate, and propionate) in MMM were prepared and were either left untreated or depleted of mucin via filtration through 3000 MWCO protein concentrators prior to analysis via reverse-phase high performance liquid chromatography (HPLC). For untreated mucin rich standards, unstable baseline readings, peak shouldering, missing peaks, and other chromatogram anomalies were observed (Fig. 2a), underscoring how mucins obfuscate analyte quantification via HPLC. In contrast, resulting chromatograms of mucin depleted standards were free from previously observed aberrations (Fig. 2b), indicating mucin depletion via PES filtration is sufficient sample preparation for HPLC organic acid profiling. To further demonstrate the efficacy this sample preparation method, a panel of eight organic acids (3.125 mM, Table 1) and eight amino acids (0.938 mM, Table 2) were prepared as mixed standards in mucin-rich MMM and subjected to the full HPLC workflow to provide a chromatogram containing peaks of all analytes of interest (Fig. 2c). These analytes were chosen based on their known generation by fermentative bacteria of the CF airways and for their associated carboxylic acid functional groups that enable detection at λ=210 nm. These data confirm analyte retention times and adequate peak separation. While there is evidence of minor peak overlap in some instances, peaks are clearly distinguishable from one another. Subsequent analysis of biological samples (Fig. 3a, c, e) suggest peak overlap is less prevalent in practice, as biological samples are less likely to contain the high concentration of analytes as in the artificial mixed standard (Fig. 2c).

Further validation of the workflow was performed by analysis of various biological samples of higher complexity than standards in a minimal medium; (i) cell-free supernatants of CF associated bacteria (*Streptococcus gordonii*, *Prevotella melaninogenica, Veillonella parvula,* and *Fusobacterium nucleatum*) grown in MMM (Figs 3a, b and S1, available in the online version of this article), (ii) cell-free supernatant of a mixed anaerobic bacterial community isolated from CF sputum (Fig. 3c, d), and (iii) a mucus-rich clinical CF sputum sample from which the enrichment was derived (Fig. 3e, f). Each sample was analysed using the described workflow, including PES filtration, and concentrations (mM) of select organic acids (Table 1) were calculated from the chromatograms. While several amino acids were detectable, PES filtration yields of this analyte group (Table 2) were variable and over 100 % in some instances. Because of this, this workflow may be better suited for detection and relative quantification of amino acids rather than absolute quantification. Consequently, we elected not to include amino acid analytes in the metabolite profiles (Fig. 3b, d, f). Data presented demonstrate the ability of this workflow to not only quantify complex biologically derived organic acid profiles from minimal defined medium, but also directly from bacterial cultures and complex human-derived material.

### Column integrity

**Fig. 4.**
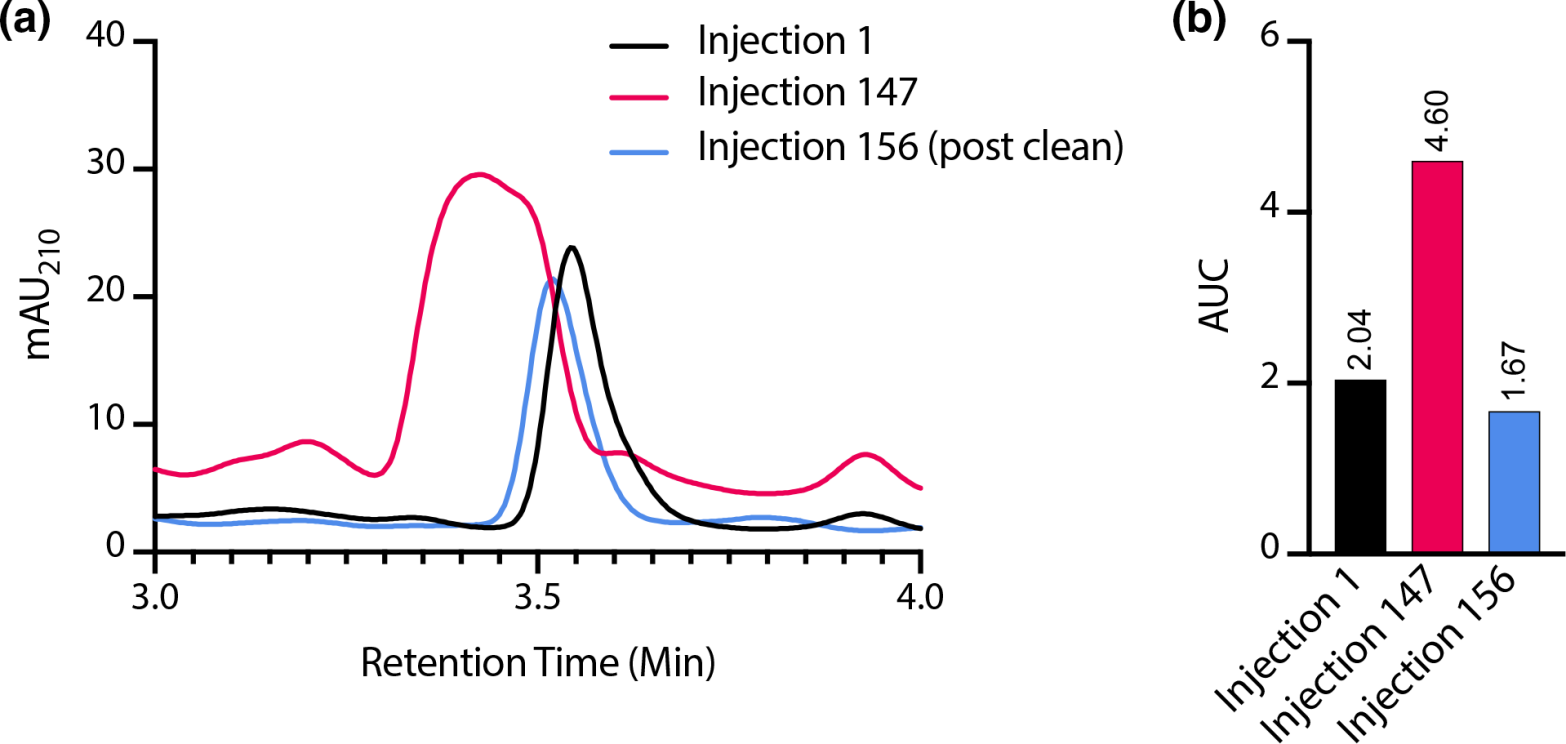
Analytical column integrity is preserved with regular maintenance. (**a**) HPLC chromatogram of the same 10 mM standard of acetate prepared in mucin-depleted MMM and run using the same method as the first, 147^th^, and 156^th^ injection on a new analytical column. Column cleaning was performed after the 155^th^ injection before the final acetate analysis. (**b**) AUC values calculated from the HPLC chromatogram.

While the data demonstrate the efficacy of our sample preparation and HPLC methods for metabolite quantification, we questioned its long-term use. More specifically, we asked whether repeated analysis of mucin-rich samples would lead to analytical column performance decline and/or damage over time. To ensure that low levels of residual low molecular weight mucins that remain after sample preparation (Fig. 1) do not significantly impact the lifespan of the column, a 10 mM standard of acetate was measured multiple times across a run of ~150 mucin-rich samples and standards of various types. As shown in Fig. 4a, deterioration of peak properties (i.e. baseline, peak width, peak height) for the acetate standard were observed after 146 consecutive runs. However, peak properties were restored after a completing a standard acetonitrile-based column cleaning protocol and subsequent equilibration. AUC values of the peaks corroborate restoration of peak resolution and area after column cleaning (Fig. 4b). Degradation of column retention and resolution with use is expected with any method as HPLC analytical columns are generally consumable products. However, data shown here affirm the sustainability of our optimized HPLC workflow, provided the column is properly maintained. For optimal column performance, we recommend a column wash protocol be completed after the analysis of each individual sample set. Replacement of the guard cartridge between sample sets of different origins (i.e. clinical samples, MMM) is also ideal. Analysis of freshly prepared standards prior to analysis of new sample set is recommended to ensure accurate peak identification and concentration calculation over the lifespan of the column. Once peak resolution is reduced and the lower range of analyte concentrations are no longer detectable, it is recommended that a new column be used.

## Conclusion

We describe a filter-based method for depletion of mucins in clinical and laboratory samples and an accompanying single-step reverse-phase HPLC method capable of organic acid quantification without the need for subsequent mass spectrometry analysis. Our data demonstrate the depletion of HMW glycoproteins, while greatly reducing the concentration of LMW mucins. This filter-based sample preparation method was also shown to reduce the loss of acetate (filter=0.06 % loss / extraction=29.0 % loss), butyrate (filter=3.6 % loss / extraction=29.4 % gain), lactate (filter=6.4 % loss / extraction=16.8 % loss), and propionate (filter=8.07 % loss / extraction=29.9 % loss) yield when directly compared to conventional liquid-liquid extraction techniques. Additionally, our sample preparation method was found to preserve integrity of the analytical column and HPLC instrument. Using this workflow, we demonstrate the successful quantification of organic acids derived from individual anaerobic bacteria (*Streptococcus gordonii*, *Prevotella melaninogenica, Veillonella parvula,* and *Fusobacterium nucleatum*), a complex mucin enrichment community derived from a clinical CF sputum sample, as well as the paired raw clinical sample itself. This approach paves the way for future studies of mucin-rich clinical samples (e.g. CF sputum, sinus mucus) in which anaerobic microbiota are widely appreciated to comprise a significant component of the microbiota, yet their contributions to disease pathogenesis are poorly understood. Through mucin depletion and accurate quantification of their mixed acid fermentation metabolites, our approach promises to generate greater insights into how they shape the host chemical environment. While we use the respiratory tract as our model system for method validation, this workflow represents a high-throughput and cost-effective method for quantification of microbial organic acids across a wide array of mucin-rich host environments.

## Supplementary material

10.1099/jmm.0.001708 Fig. S1.

## Abbreviations

BHI: brain heart infusion
CF: cystic fibrosis
CFTR: cystic fibrosis transmembrane conductance regulator
FPLC: fast protein liquid chromatography
GI: gastrointestinal
HMW: high molecular weight
HPLC: high-performance liquid chromatography
MMM: minimal mucin media/medium
MWCO: molecular weight cutoff
OA: organic acid
PES: polyethersulfone
RC: regenerated cellulose
RS: rapid separation
