# Supplementary material for: High-throughput quantification of microbial-derived organic acids in mucin-rich samples via reverse phase high performance liquid chromatography
Source: J Med Microbiol. 2023 Jun 9;72(6):001708. doi: 10.1099/jmm.0.001708 (PMC13293341; doi:10.1099/jmm.0.001708)
Supplement: Fig. S1. [file jmm-72-01708-s001.pdf]

Supplemental Data

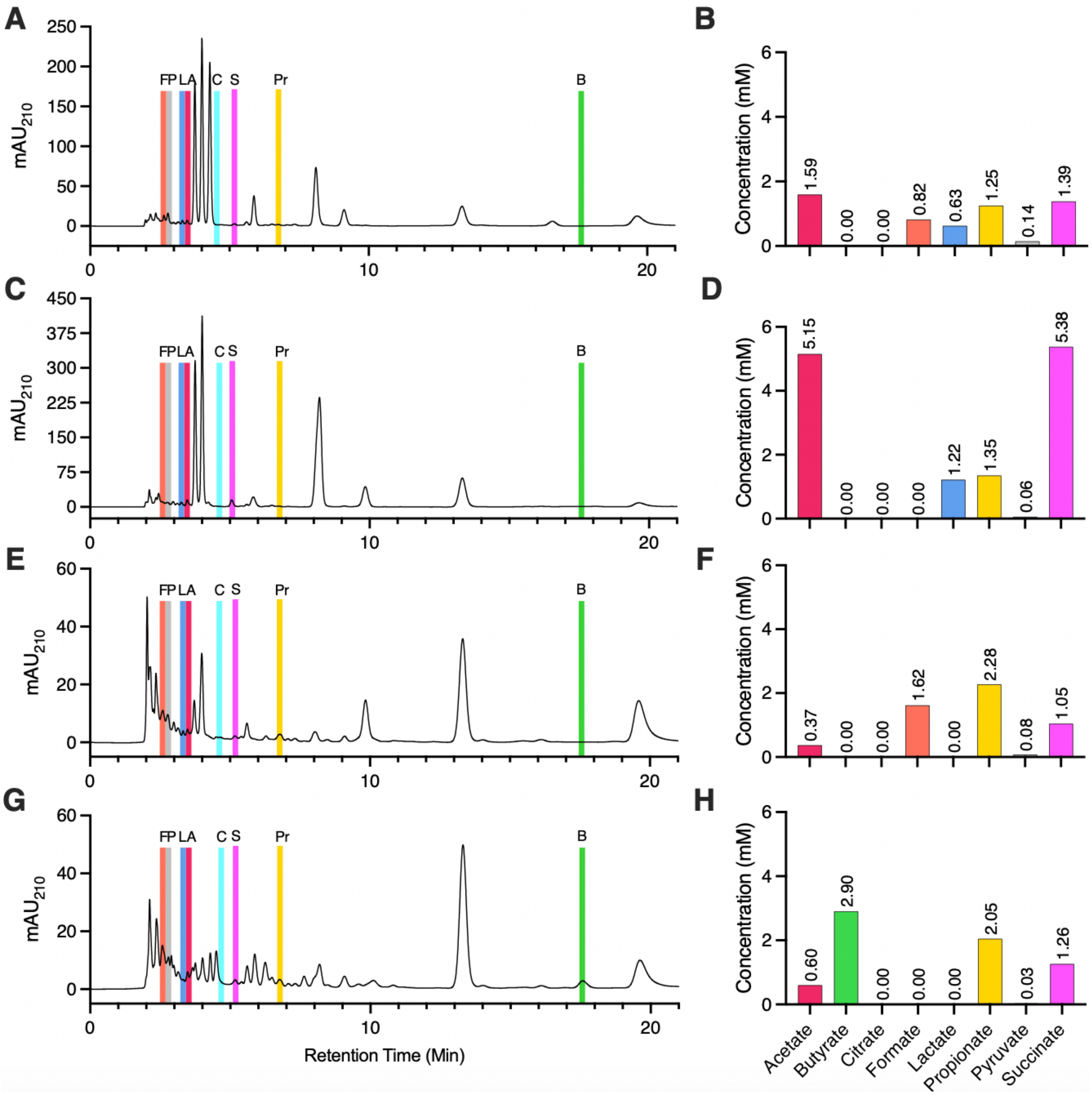

Figure S1. HPLC-based quantification of organic acids in mucin-rich cell free supernatants derived from cystic fibrosis associated microbiota. HPLC chromatogram and corresponding organic acid profile of supernatants generated by **A,B)** *Prevotella melaninogenica*, **C,D**) *Veillonella parvula*, and **E,F)** *Fusobacterium nucleatum*.
